# Proteomics analysis of cancer tissues identifies IGF2R as a potential therapeutic target in laryngeal carcinoma

**DOI:** 10.3389/fendo.2022.1031210

**Published:** 2022-10-10

**Authors:** Bing Liu, Yuqiang Hu, Lixia Wan, Luan Wang, Liangjun Cheng, Hai Sun, Yaran Liu, Di Wu, Jiefei Zhu, Xiu Hong, Yang Li, Chong Zhou

**Affiliations:** ^1^ Xuzhou Clinical School, Xuzhou Medical University, Xuzhou, China; ^2^ Department of Otolaryngology-Head and Neck Surgery, Xuzhou Central Hospital, Xuzhou, China; ^3^ Department of Psychology, Xuzhou Central Hospital, Xuzhou, China; ^4^ Institute of Medical Artificial Intelligence, Binzhou Medical College, Yantai, China; ^5^ Department of Pathology, Xuzhou Central Hospital, Xuzhou, China; ^6^ Central laboratory, Xuzhou Central Hospital, Xuzhou, China; ^7^ Department of Radiation Oncology, Xuzhou Central Hospital, Xuzhou, China

**Keywords:** proteomics, laryngeal cancer, IGF2R (receptor of insulin growth factor 2), TCGA, target therapeutic

## Abstract

**Background:**

Laryngeal cancer (LC) is a prevalent head and neck malignancy; however, the essential pathophysiological mechanism underlying its tumorigenesis and progression remains elusive. Due to the perduring scarcity of effective targeted drugs for laryngeal cancer, insights into the disease’s pathophysiological mechanisms would substantially impact the treatment landscape of laryngeal cancer.

**Methods:**

To ensure quality consistency, 10 tumor and 9 non-tumor samples underwent proteomic analysis on a single mass spectrometer using a label-free technique. Subsequently, gene expression variations between laryngeal squamous cell carcinoma and normal tissues were analyzed using The Cancer Genome Atlas (TCGA) database. Immunohistochemical expressions of insulin-like growth factor 2 receptor (IGF2R), fibronectin (FN), vimentin, and α-smooth muscle actin (SMA) in LC tissues and normal tissues were determined.

**Results:**

In the tumor group, significant variations were detected for 433 upregulated and 61 downregulated proteins. Moreover, the heatmap revealed that the expressions of RNA translation-related proteins and proteins involved in RNA metabolism, such as IGF2R, tenascin C (TNC), periostin (POSTN), proteasome 26S subunit ATPase 4 (PSMC4), serpin family A member 3 (SERPINA3), heat shock protein family B (small) member 6 (HSPB6), osteoglycin (OGN), chaperonin containing TCP1 subunit 6A (CCT6A), and chaperonin containing TCP1 subunit 6B (CCT6B), were prominently elevated in the tumor group. Nonsense-mediated RNA decay (NMD), RNA translation, and protein stability were significantly altered in LC tumors. IGF2R was remarkably upregulated in LC tumors. In the TCGA database, the IGF2R mRNA level was significantly upregulated in LSCC tissues. Additionally, IGF2R mRNA expression was lowest in clinical grade 1 samples, with no significant difference between grades 2 and 3. In LSCC patients, a significant positive correlation between IGF2R expression and the stromal score was detected using the ESTIMATE algorithm to estimate the immune score, stromal score, and tumor purity in the tumor microenvironment. Lastly, immunohistochemical analysis revealed that IGF2R is overexpressed in LC.

**Conclusion:**

These results demonstrate the vital role of IGF2R in LC carcinogenesis and progression and may facilitate the identification of new therapeutic targets for the prevention and treatment of LC.

## Introduction

Laryngeal cancer (LC) is one of the most commonly encountered head and neck cancers and a major cause of death worldwide (1). In recent decades, the 5-year survival rate for LC has improved due to the use of comprehensive treatment modalities such as surgery, radiotherapy, chemotherapy, and immunotherapy (2–4). Early diagnosis of LC is critical for long-term survival, the clinical outcome being strongly dependent on the cancer’s stage at the time of diagnosis (5). Consequently, there is a pressing need to investigate the mechanistic factors governing the genesis and progression of LC and to identify critical biomarkers in order to develop new preventative and therapeutic interventions.

Over the past few decades, immunohistochemistry has been extensively utilized in laryngeal cancer research. However, high-throughput protein detection is not feasible *via* immunohistochemistry. The rapid expansion of proteomics has shed light on the mechanism underlying tumorigenesis and development. Variations in protein abundance between tumors and normal tissue can be interpreted as tumor-specific alterations. Identifying key molecules that influence laryngeal cancer development will contribute to the future development of tumor-specific targeted drugs. Literature (6) also suggests that genes or signaling pathways regulating cell proliferation may be associated with accelerated tumor progression and a poor prognosis; notable examples include the Notch pathway, hypoxia-inducible factor 1-alpha (HIF1-α), and epidermal growth factor receptor (EGFR). Ren, K et al. (7) reported that EGFR antibody antagonist 6E-C inhibited laryngeal cancer *in vitro* and *in vivo*.

There are currently few literature reports on the application of proteomics techniques in head and neck tumors (8, 9), and protein heterogeneity in tumor tissues reflects the biological heterogeneity of tumors. Liu et al. (8) conducted a proteomic comparison of 10 pairs of cancerous and normal mucosal samples from patients with advanced head and neck cancer who shared similar clinical features. Their results revealed that 240 differentially expressed proteins were enriched in molecular networks regulating cytoskeleton remodeling and antigen presentation. Astradsson, T et al. (10) probed the expression of inflammatory proteins in serum using proteomics on samples from patients with head and neck cancer pre and post-chemoradiation treatment and discovered a significant correlation between serum inflammatory proteins and treatment modality.

Few studies have employed high-throughput proteomics techniques to analyze the mechanistic factors driving the occurrence and progression of laryngeal cancer. To address these questions, we first utilized proteomics for a comparative analysis of protein expression in laryngeal cancer and paracancerous normal tissues. Secondly, additional analyses were performed based on The Cancer Genome Atlas (TCGA) datasets. Subsequently, biomarker candidates for IGFR2 were selected from the protein list derived by the above methods and validated by immunohistochemical analysis of clinically-confirmed LC samples.

## Method

### Patient selection and ethical approval

For the proteomics analysis, 10 patients with biopsy-confirmed LC were selected from patients who underwent surgical biopsy in the Department of Otolaryngology of Xuzhou Central Hospital between 2020.11 and 2022.2. The inclusion criteria encompassed (1): Laryngeal cancer T2-T3N0M0 without invasion of other sub-regions, such as the base of the tongue and the hypopharynx. (2) Patients without previous surgery or chemoradiation. (3) The absence of additional malignant tumors beyond laryngeal cancer. (4) Patients without diabetes. (5) Availability of clinical and laboratory information at the time of diagnosis. The study protocol was approved by the Ethics Committee of Xuzhou Central Hospital. Informed consent was obtained from all patients enrolled in the proteomic analysis. The study was performed in accordance with the Declaration of Helsinki. The clinical characteristics of enrolled patients are summarized in **Table S1**.

### Proteomics analysis

Protein Extraction: 10 frozen LC tissue samples were processed. Blood was washed away using pre-cooled saline solution. The tissues were ground with liquid nitrogen, and after the addition of Roche’s lysate (at approximately 7 times the weight, mL), broken by ultrasound. Finally, the mixture was centrifuged at 15,000 g for 15 min and the supernatant was collected for protein concentration measurements.

Enzymatic Hydrolysis: Acetone (4 times the sample volume) was added to the supernatant for protein precipitation. The protein was re-dissolved using 8 M urea. Prior to enzymatic digestion of the protein, trichloroethyl phosphate (TCEP) and chloroacetamide were used to open the disulfide bonds and protect the thiol. Lastly, trypsin was added to the protein at 50:1 for an enzymatic reaction over 16 hours to obtain the peptide.

LC-MS Analysis Method: Peptide isolation was performed using AB Nano-LC 400 (AB SCIEX, USA) with a 150 mm by 100 mm AquaC18 column, and peptide analysis was performed using the AB Triple TOF 5600 Mass Spectrometer (AB SCIEX, USA). The solvent was ACN (solution A) and water containing 0.1% acetic acid (solution B). The HPLC gradient was 5% to 80% of solution A after 90 min with a flow rate of 1,000 nL/min.

### Collection of gene expression data and expression difference analysis

Gene expression profiles and clinical data for 111 LSCC tumor and 12 normal tissues were retrieved from the TCGA database (accessible via: https://portal.gdc.cancer.gov/). Fragments per kilobase of exon per million fragments mapped (FPKM) were extracted as a measure of gene expression. The Student t-test was applied to evaluate the expression difference between tumor and normal tissues. Moreover, gene expression differences across multiple clinical grades were assessed.

### Evaluation of the relationship between gene expression and the tumor microenvironment, immune cell infiltration, and immune checkpoint molecules

Estimation of Stromal and Immune cells in MAlignant Tumor tissues using Expression data (ESTIMATE) is an algorithm for estimating the immune score, stromal score, and tumor purity within the tumor microenvironment. In the present study, the immune score, stromal score, and tumor purity score were calculated using the ESTIMATE algorithm. The relationship between gene expression and tumor microenvironment was assessed using a Pearson correlation analysis.

Tumor Immune Estimation Resource (TIMER, accessible via: https://cistrome.shinyapps.io/timer/) is a comprehensive resource for analyzing and visualizing the abundances of tumor-infiltrating immune cells, including macrophages, neutrophils, dendritic cells, CD4+ T cells, CD8+ T cells, and B cells. CIBERSORT (https://cibersort.stanford.edu/) is an analytical tool that uses gene expression data to estimate the abundance of 22 constituent cell types in a mixed cell population. Estimations of immune cell infiltration abundance for LSCC patients were derived *via* TIMER and CIBERSORT. Using the median gene expression as a threshold, LSCC patients were divided into two groups to investigate differences in immune cell infiltration.

Eight crucial immune checkpoint molecules were retrieved from the literature, namely prephenate dehydratase 1 (PD-1), programmed cell death 1 ligand 1 (PD-L1), programmed cell death 1 ligand 2 (PD-L2), T cell immunoreceptor with Ig and ITIM domains (TIGIT), lymphocyte activating 3 (LAG3), hepatitis A virus cellular receptor (HAVCR2), sialic acid binding Ig-like lectin 15 (SIGLEC15), and cytotoxic T-lymphocyte associated protein 4 (CTLA4). To investigate the relationship between gene expression levels, a Spearman rank correlation analysis was conducted.

### Immunohistochemistry stain

For IHC, formalin-fixed paraffin-embedded (FFPE) tissues collected from 7 patients with LC were used. Clinical and pathological information on the patients is displayed in **Table S2**.

IHC was performed according to standard procedures. 5 μm thick FFPE human tissue sections were utilized for the experiments. Used antibodies include α-SMA (1:1000, Servicebio, GB13044), vimentin (1:1000, Servicebio, GB11192), IGF2R (1:200, Wuhan Sanying, 20253-1-AP), and FN (1:300, Servicebio, GB13091). Stained slides were scanned using a Panoramic MIDI with a 40× objective lens. Hematoxylin-stained nuclei were blue, whereas DAB-positive expression was brownish yellow.

### Statistical analysis

MaxQuant software (version 1.6.17.0) embedded with the Andromeda search engine was utilized to conduct a bioinformatic analysis. Raw LC-MS/MS files were searched against the UniProt human proteome database (11). Up to two missed cleavages were permitted. Carbamidomethyl (C) was considered as a fixed modification, whereas oxidation (M) and N-terminal acetylation were variable modifications. The false discovery rate (FDR) threshold was set as 0.01 for both proteins and peptides. The student’s t test was used to evaluate variations in protein expression between the study groups. The R clusterprofiler (version 3.16.1) package was used to implement a pathway enrichment analysis (12). Protein-protein functional networks were constructed using the default settings and visualized with the Cytoscape (version 3.8.1) software (13).

## Results

### Proteomics data identifies IGF2R as a potential candidate in LC

**Figure 1:**
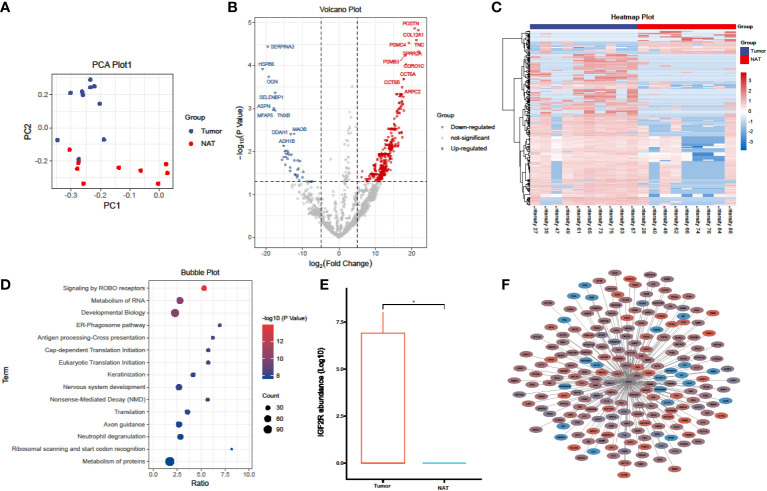
Proteomics analysis comparing laryngeal cancer to adjacent normal tissues. **(A)** PCA plots of the proteins identified in laryngeal cancer and adjacent normal tissues (laryngeal cancer tissue, n = 10; adjacent normal tissues, n = 9). **(B)** Volcano plot showing the DEPs in laryngeal cancer and adjacent normal tissues. **(C)** A heatmap illustrating the expression of DEPs in laryngeal cancer and adjacent normal tissues; each row represents one protein, and each column represents one sample. **(D)** A bubble plot depicting the enriched KEGG pathways of DEPs identified in laryngeal cancer and adjacent normal tissues. **(E)** A box plot of IGF2R protein expression in laryngeal cancer and adjacent normal tissues. *p < 0.05, student’s t test. **(F)** Network analysis of the correlation between DEPs and IGF2R (|R| > 0.5, P < 0.05), line thickness represents the strength of the correlation, blue represents downregulation, and red represents upregulation.

Mass spectrometry (MS)-based proteomics can provide more profuse biological insights than genomic analysis alone, owing to its capacity for measuring global protein abundance. Combining sequencing and MS provides a more comprehensive picture of the association between cancer “genotype” and “phenotype” *via* functional proteomics and signaling networks. Thus, we paired surgically-resected primary tumor tissues and non-tumor larynx tissues from 10 LC patients without prior chemotherapy or radiotherapy. Proteomic analysis was performed on a single mass spectrometer using a label-free technique to assure quality consistency. 2,608 proteins were identified from 10 tumor and 9 non-tumor samples (one non-tumor tissue was omitted after failing the QC stage). Principal component analysis demonstrated marked differences between the proteomes of tumor and non-tumor samples, highlighting the high heterogeneity among tumor samples (**Figure 1A**). This was followed by a differentially expressed proteins (DEPs) analysis between tumor and non-tumor samples. As indicated by the volcano plot, significant alterations in the tumor group encompassed 433 upregulated and 61 downregulated proteins (**Figure 1B**, P< 0.05 and fold change > 2 or < 0.5). The complete DEPs list can be found in **Supplementary Table S2**). The heatmap demonstrated that RNA translation-related and RNA metabolism-related proteins, including IGF2R, TNC, POSTN, PSMC4, SERPINA3, HSPB6, OGN, CCT6A, and CCT6B, were conspicuously upregulated in tumor group (**Figure 1C**).

A subsequent pathway enrichment analysis revealed that NMD RNA decay, RNA translation, and protein stability were significantly altered in LC tumors (**Figure 1D**), which corroborates the human LC transcriptome data analysis. Of all identified candidate proteins, insulin-like growth factor 2 receptor (IGF2R) was markedly upregulated in LC tumors (**Figure 1E**). We further constructed the protein-protein network of IGF2R and its correlated DEPs (|R| > 0.5, P < 0.05) (**Figure 1F**); the network demonstrated that numerous proteins involved in RNA translation and protein metabolism were co-upregulated in LC tumors. Therefore, our proteomics data provides evidence that IGF2R holds a vital role in LC carcinogenesis and progression.

### IGF2R is significantly upregulated in LSCC tissues

**Figure 2:**
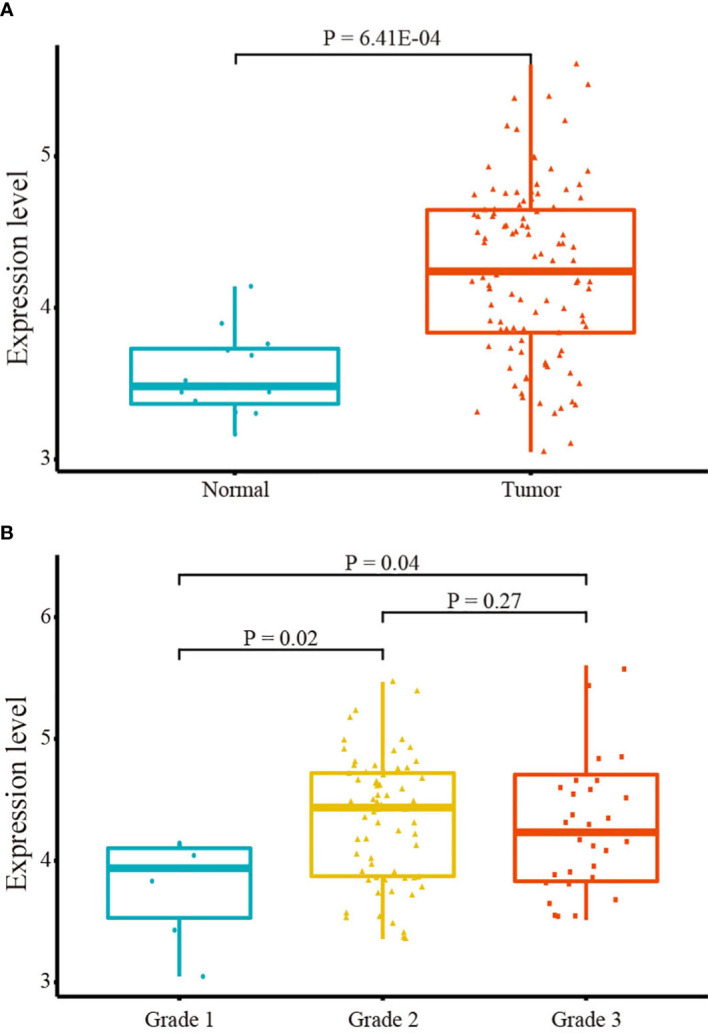
IGF2R mRNA expression. **(A)** IGF2R expression in tumor and normal tissues. **(B)** IGF2R expression across clinical grades.

The student t-test was employed to compare IGF2R expression between LSCC and normal tissues in the TCGA database. IGF2R was significantly upregulated in LSCC tissues (**Figure 2A**). Furthermore, differences in expression were observed across different clinical grades. As depicted in **Figure 2B**, IGF2R levels were lowest in the clinical grade 1 phase, indicating a potential correlation between an increased IGF2R mRNA level and LSCC progression. However, no significant difference in IGF2R expression was observed between Grades 2 and 3.

### Relationship between IGF2R expression and the tumor microenvironment, immune cell infiltration, and immune checkpoint molecules

**Figure 3:**
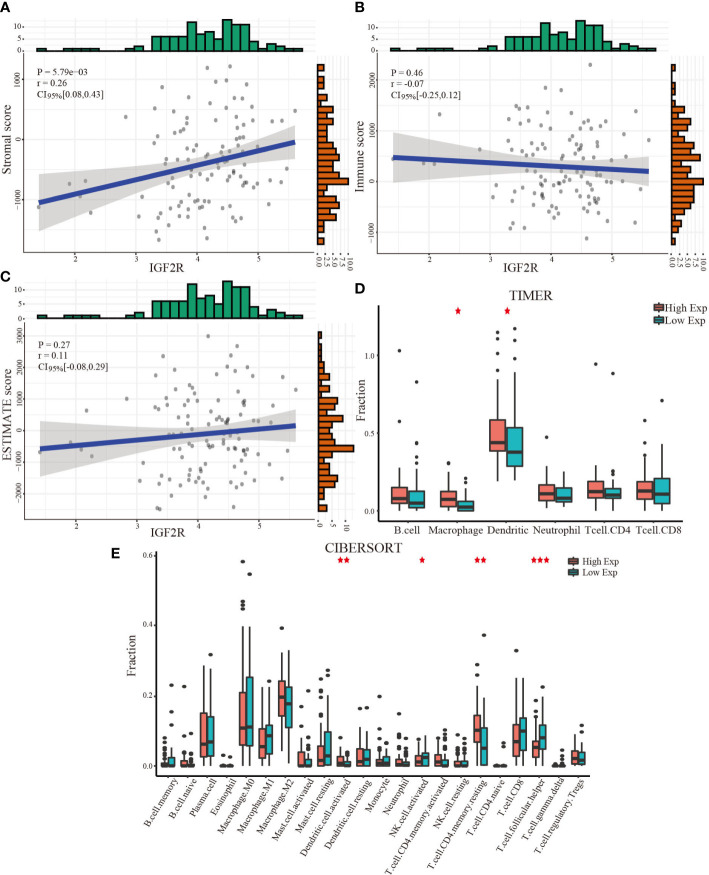
Relationship between IGF2R expression and the tumor microenvironment and immune cell infiltration. **(A)** IGF2R expression and the stromal score. **(B)** IGF2R expression and the immune score. **(C)** IGF2R expression and the ESTIMATE score. **(D)** Levels of six infiltrating immune cells across IGF2R expression as per TIMER. **(E)** Levels of 22 subtypes of infiltrating immune cells across IGF2R expression as per CIBERSORT. *,**,*** represent P<0.05,0.01,0.001.

Using the ESTIMATE algorithm to evaluate the immune score, stromal score, and tumor purity in the tumor microenvironment of LSCC patients based on the TCGA database, we conducted a Pearson correlation analysis to ascertain the relationship between gene expression and the tumor microenvironment. As illustrated in **Figures 3A–C**, a significant positive correlation between IGF2R expression and the stromal score was observed (r = 0.26, P = 5.79E-03). However, immune score and ESTIMATE score data revealed no significant correlations.

**Figure 4:**
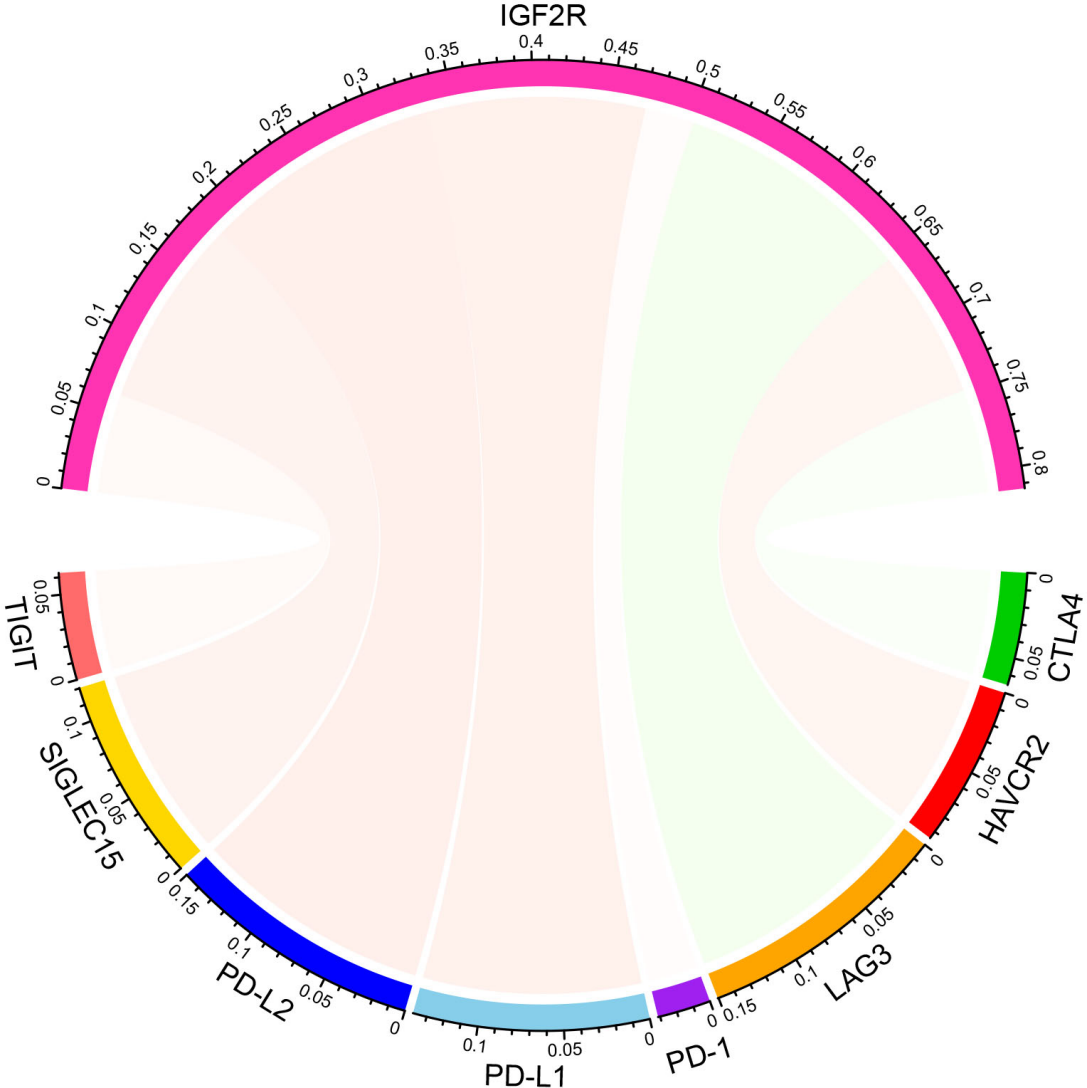
Correlations between expressions of IGF2R and eight important immune checkpoint molecules.

We utilized the TIMER and CIBERSORT databases to uncover the association between gene expression and immune cell infiltration. Compared with the low IGF2R expression samples, macrophages and dendritic cells were significantly enriched in the high-IGF2R expression samples (**Figure 3D**). As illustrated in **Figure 3E**, patients with high IGF2R expression had significantly higher proportions of activated dendritic cells and resting memory CD4+ T cells, but significantly lower proportions of activated natural killer (NK) cells and follicular helper T cells. Additionally, we investigated whether a correlation existed between IGF2R gene expression and eight important immune checkpoint molecules. As illustrated in **Figure 4**, IGF2R expression correlated positively with PD-L1, PD-L2, and SIGLEC15 expressions in the TCGA-LSCC dataset, whereas it correlated negatively with LAG3 expression.

### Immunohistochemical validation of IGF2R expression in LC and paracancerous samples

**Figure 5:**
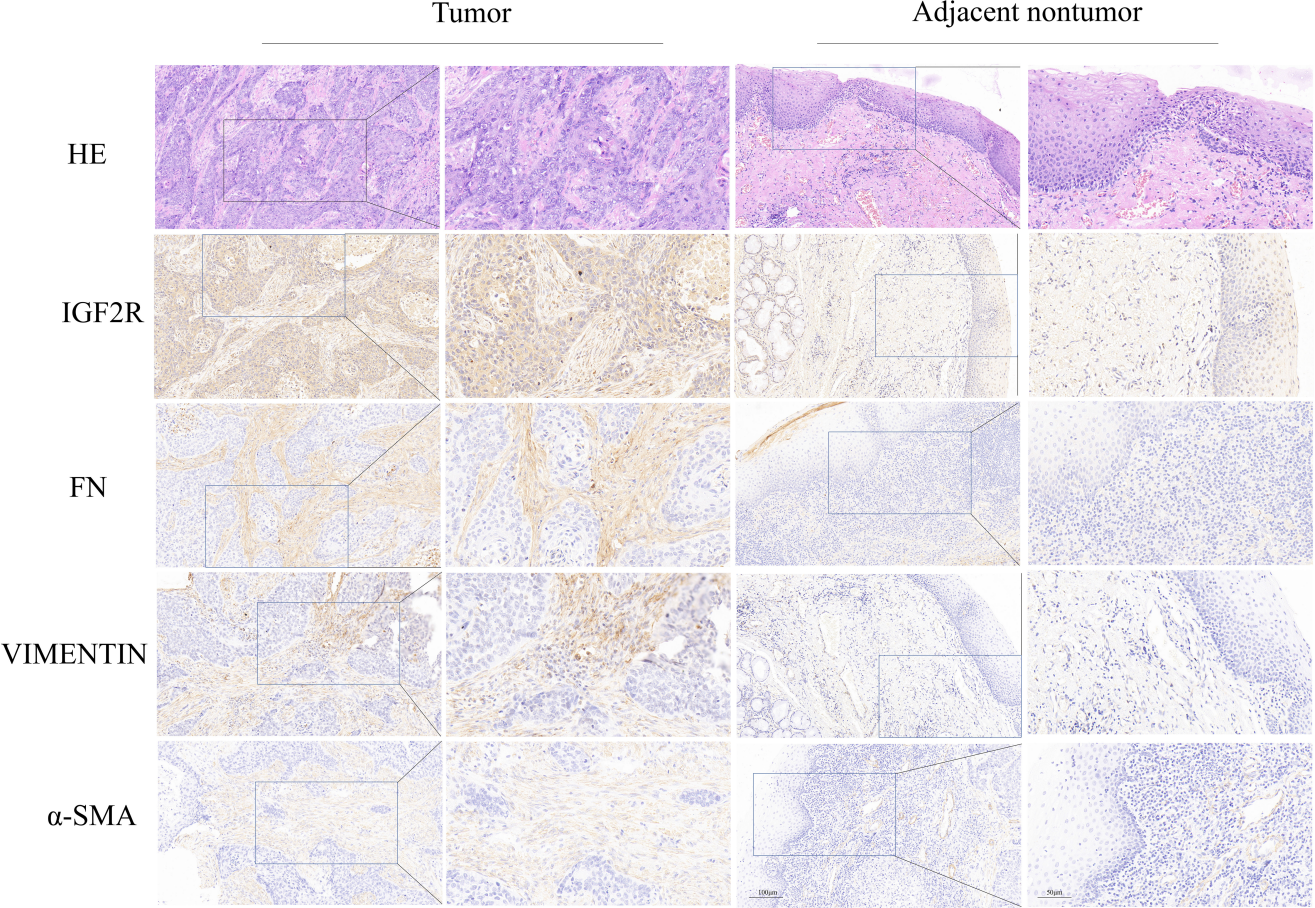
Immunohistochemical expression of IGF2R, FN, Vimentin, and SMA in LC and normal tissues.

HE staining revealed that laryngeal cancer cells exhibit an infiltrative growth pattern with adjacent interstitial fibrous tissue reactions. IGF2R was expressed in the cytoplasm or nuclei of squamous cell carcinoma cells but not in the normal squamous epithelium. FN, Vimentin, and SMA were significantly overexpressed in the interstitial fibrous tissue and myofibroblasts surrounding cancerous tissue compared to the normal squamous epithelium (**Figure 5**).

## Discussion

Laryngeal cancer is among the prevailing head and neck malignancies; however, the key pathophysiological mechanism underlying its tumorigenesis and progression remains elusive. We demonstrated that IGF2R was conspicuously upregulated in LC tumors and validated our results by revealing the correlations between IGF2R expression and the tumor microenvironment and immune cell infiltration. Additionally, we confirmed that IGF2R was overexpressed in LC *via* IHC.

Among existing proteomic analysis-related research articles, reports exclusively addressing LC are scarce. Burian A et al. (14) determined that 8 proteins were significantly more abundant and 9 were downregulated in formalin-fixed paraffin-embedded tissue samples from tumors. Our study differs in its use of fresh surgical specimens, which theoretically diminishes protein degradation to the fullest extent and ensures the accuracy of the results. In this study, 433 proteins were significantly upregulated, and 61 proteins were significantly downregulated in the tumor group compared to adjacent normal tissue. Among all identified biomarker candidates, IGF2R was markedly upregulated in LC tumors. According to our knowledge, we were the first to discover that the IGF2R protein is abnormally expressed in laryngeal cancer tissue.

Several studies have uncovered a positive association between IGF2R expression levels and the risk of various tumors (15, 16). Another study has demonstrated that IGF2R overexpression enhanced the suppressive effect of irradiation on cancer cell viability and invasiveness in lung adenocarcinoma cells, whereas this effect was attenuated by IGF2R knockdown (17). Through an integrated analysis of transcriptional profiles, our study confirmed that IGF2R was upregulated in LSCC tissues. Furthermore, we discovered IGF2R expression variations between clinical grades. Such results are indicative of a potential role of IGF2R as an oncogene associated with LSCC progression.

In recent years, numerous studies have demonstrated that the tumor microenvironment, including immune cells, is a major contributor to tumor progression that can modulate inflammation and metastasis in myriad ways (18–20). In this study, a systematic evaluation was conducted to define the relationship between IGF2R expression and the tumor microenvironment, immune cell infiltration, and immune checkpoint molecules. Chen et al. reported that Trop2 binding to IGF2R upregulated the IGF2-IGF1R-Akt axis to enhance resistance to gefitinib and remodeling of the tumor microenvironment in NSCLC (21). In our study, a significant correlation was observed between IGF2R expression and the stromal score, indicating a potential regulatory effect of IGF2R on the tumor microenvironment and LSCC progression. Using the TIMER database, we also uncovered that macrophages and dendritic cells were significantly enriched in samples with high IGF2R expression.

Wang et al. discovered that IGF2R activation promoted proton rechanneling to the mitochondrial intermembrane space and enabled sustained oxidative phosphorylation, outlining a previously unidentified role of IGF2R activation in modulating anti-inflammatory macrophages (22). The present study’s expression correlations analysis revealed that IGF2R expression correlated significantly with multiple immune checkpoint molecules (including PD-L1, PD-L2, SIGLEC15, and LAG3), supporting the use of IGF2R as a novel biomarker for determining the efficacy of immunotherapy. Ramakrishnan et al. found that IGF2R functions as a receptor for GZMB/GrzB and may mediate cell killing. Their results demonstrated that the effect of immunotherapy is significant only when increased levels of IGF2R are present on the surface of tumor cells (23).

To confirm that IGF2R is overexpressed in laryngeal cancer compared with paracancerous normal tissues, our study also included immunohistochemical staining. Protein expression levels were assessed for α-SMA, Vimentin, and FN, markers of tumor aggressiveness associated with epithelial-mesenchymal transition (EMT) (24). IGF2R expression was consistent with the expression pattern of these biomarkers, suggesting that IGF2R may potentially promote tumor EMT *via* the IGF2/IGF2R signaling pathway.

This study has certain limitations. Firstly, a small sample size was included in the proteomics analysis; hence, the study is not suitable for further stratification of the results based on clinical pathological characteristics. Secondly, IGF2R was not studied *in vitro*; hence, there is presently no in-depth comprehension of IGF2R’s role in laryngeal cancer invasion and metastasis. Nevertheless, this preliminary analysis demonstrates the involvement of IGF2R molecules in the tumorigenesis of LC and lays the groundwork for further research on IGF2R in the context of LC.

## Conclusion

This study’s findings indicate that IGF2R plays a significant role in LC carcinogenesis and progression and may support the identification of new therapeutic targets for the prevention and treatment of LC.

## Data availability statement

The datasets presented in this study can be found in online repositories. The names of the repository/repositories and accession number(s) can be found below: https://www.ncbi.nlm.nih.gov/, https://portal.gdc.cancer.gov.

## Ethics statement

The study protocol was approved by the Ethics Committee of Xuzhou Central Hospital. The patients/participants provided their written informed consent to participate in this study.

## Author contributions

CZ, YL, and BL conceived and designed the study. LXW, LW, HS, YLR, DW, JZ, XH, and LJC were responsible for data collection, analysis, and verification. BL wrote the initial draft of the manuscript, which was reviewed and revised by CZ and YL. All authors contributed to the article and approved the submitted version.

## Funding

This work was funded by the Jiangsu Provincial Health Commission project (No. Z2021016).

## Conflict of interest

The authors declare that the research was conducted in the absence of any commercial or financial relationships that could be construed as a potential conflict of interest.

## Publisher’s note

All claims expressed in this article are solely those of the authors and do not necessarily represent those of their affiliated organizations, or those of the publisher, the editors and the reviewers. Any product that may be evaluated in this article, or claim that may be made by its manufacturer, is not guaranteed or endorsed by the publisher.
