# Supplementary material for: Proteomics analysis of cancer tissues identifies IGF2R as a potential therapeutic target in laryngeal carcinoma
Source: Front Endocrinol (Lausanne). 2022 Oct 10;13:1031210. doi: 10.3389/fendo.2022.1031210 (PMC9592118; doi:10.3389/fendo.2022.1031210)
Supplement: Supplementary file 1 [file Table_1.docx]

Table s1. Clinical and pathological information of patients in proteomics

| Age(y) | Gender | Stage | Grade |
| --- | --- | --- | --- |
| 78 | Male | T1N0M0 | 1 |
| 67 | Male | T2N0M0 | 2 |
| 51 | Male | T1N0M0 | 1 |
| 61 | Male | T1N0M0 | 1 |
| 54 | Male | T1N0M0 | 2 |
| 43 | Male | T1N0M0 | 3 |
| 66 | Male | T2N0M0 | 2 |
| 61 | Male | T2N0M0 | 1 |
| 70 | Male | T1N0M0 | 1 |
| 75 | Male | T1N0M0 | 2 |
