# Supplementary material for: Proteomics analysis of cancer tissues identifies IGF2R as a potential therapeutic target in laryngeal carcinoma
Source: Front Endocrinol (Lausanne). 2022 Oct 10;13:1031210. doi: 10.3389/fendo.2022.1031210 (PMC9592118; doi:10.3389/fendo.2022.1031210)
Supplement: Supplementary file 2 [file Table_2.docx]

Table s2. Clinical and pathological information of patients in Immunohistochemistry stain

| Age(y) | Gender | Stage | Grade |
| --- | --- | --- | --- |
| 68 | Male | T2N0M0 | 2 |
| 57 | Male | T1N1M0 | 3 |
| 63 | Male | T1N0M0 | 1 |
| 61 | Male | T2N0M0 | 2 |
| 73 | Male | T2N0M0 | 2 |
| 80 | Male | T3N0M0 | 3 |
| 76 | Male | T2N0M0 | 2 |
